# Mindfulness shapes emotion regulation in non-clinical adolescents: Secondary outcomes of a randomized controlled trial

**DOI:** 10.1177/13591045251377898

**Published:** 2025-09-24

**Authors:** Erik Mendola, Ben Meuleman, Mariana Magnus Smith, Vladimira Ivanova, Eléonore Pham, Valentine Savary, Patricia Cernadas, Zeynep Celen, Luigi Francesco Saccaro, Andrea Samson, Paul Klauser, Arnaud Merglen, Camille Marie Piguet

**Affiliations:** 1 Child and Adolescent Psychiatry Division, Geneva University Hospitals, Geneva, Switzerland; 2 Department of Psychiatry, Faculty of Medicine, University of Geneva, Geneva, Switzerland; 3 Division of General Pediatrics, Geneva University Hospitals & Faculty of Medicine, University of Geneva, Geneva, Switzerland; 4 Adult Psychiatry Division, Geneva University Hospitals, Geneva, Switzerland; 5 Swiss Center for Affective Sciences, University of Geneva, Switzerland; 6 Faculty of Psychology, UniDistance Suisse, Brig, Switzerland; 7 Institute of Special Education, University of Fribourg, Fribourg, Switzerland; 8 Centre for Psychiatric Neuroscience, Department of Psychiatry, Lausanne University Hospital, Lausanne, Switzerland; 9 Service of Child and Adolescent Psychiatry, Department of Psychiatry, Lausanne University Hospital, Lausanne, Switzerland

**Keywords:** Mindfulness, early intervention, adolescents, emotion regulation, randomized controlled trial, non-clinical

## Abstract

Adolescence, characterized by both remarkable brain plasticity and vulnerability to psychiatric disorders; represents a uniquely propitious time window for targeted early interventions. Mindfulness-based interventions (MBI) have garnered increasing interest as cost-efficient, non-invasive and non-pharmacological approaches to enhancing mental health. While solid evidence supports the mental health benefits of MBI in adults, results in adolescents remain mixed. In particular, there is contradictory evidence regarding the use of MBI for healthy adolescents, underscoring the need for further research on its underlying mechanisms. One emerging mechanism mediating the beneficial effects of MBI is improved emotion regulation. Indeed, impaired emotion regulation is increasingly recognized as an early and transdiagnostic marker for psychiatric disorders. Therefore, we aimed to examine the impact of MBI on emotion regulation (ER) strategy use in healthy young adolescents. This study is a two-arm, wait-list, randomized controlled trial (RCT) of an 8-week MBI involving 70 adolescents from a non-clinical sample, aged 13 to 15. This study reports secondary outcomes on the impact of the MBI on emotion regulation strategy (ERS) use during a naturalistic task of emotion reactivity and regulation. Post-hoc *t*-tests on a multilevel logistic regression model (GLMM) revealed a significant decrease in the use of acceptance and problem solving, and a highly significant increase in the use of relaxation in adolescents’ emotion regulation strategies after the MBI, compared to the wait-list controls. Despite the limited sample size, these secondary results of the study point to the effectiveness of MBI in improving emotion regulation strategies in adolescents from the general population, paving the way to future wider-scale research into specific mechanisms of action and into the clinical relevance of MBI in adolescents. Shedding light on these points is pivotal for validating MBI as a potential early intervention aimed at improving mental health and reducing the risk of psychiatric disorders in adolescents.

## Plain language summary

Adolescence is a time when the brain is changing a lot, and young people are more likely to develop mental health problems. Finding ways to help teens manage their emotions early on is very important. One way to do this is through mindfulness-based interventions (MBI). These are simple, low-cost exercises that help people pay attention to the present moment and handle stress better. While mindfulness has been shown to help adults feel better mentally, it is not yet clear how well it works for teenagers, especially those who do not have any diagnosed mental health problems. This study, called the Mindfulteen Study, looked at 70 healthy adolescents aged 13 to 15. The teens were split into two groups, with one group taking part in an 8-week mindfulness program, and the other group waiting to do the program later. The study focused on how the mindfulness program affected the ways teens manage their emotions during everyday situations. After the program, teens who did mindfulness used fewer strategies like acceptance and problem-solving but used relaxation much more often to cope with their feelings. This suggests that mindfulness training helped them find new, effective ways to handle their emotions. Although the number of participants was small, the results are promising. They show that mindfulness could be a useful tool to improve emotional health in teenagers before any problems develop. More research with larger groups is needed to confirm these findings and to see if mindfulness could become a common early approach to support teen mental health.

## Introduction

Mental health is an increasingly serious global concern, particularly among youth, due to its impact on vocational training, self-esteem, family dynamics, and relationships during this developmental stage. Most psychiatric diseases emerge in adolescence and young adulthood (Solmi et al., 2022). Thus, adolescence, characterized by brain plasticity and vulnerability to psychiatric disorders, is a critical time for targeted early interventions (Romeo, 2010). During this period, the refinement of affective control abilities necessary for effective emotion regulation strategies (ERS) occurs, likely through the maturation of cortico-limbic connectivity (Schweizer et al., 2020). With prefrontal regions being the last to mature by the end of the third decade of life, this dynamic explains the relative immaturity in affective control during adolescence (Blakemore, 2019; Casey et al., 2019). While this can lead to non-pathological traits such as increased emotional intensity, impulsivity, and risk-taking, it also presents a vulnerability factor. Therefore, early interventions focused on adolescents’ mental health may help mitigate the risk or consequences of full-blown psychiatric disorders (McGorry & Nelson, 2016).

The current framework for at-risk mental states encompasses transdiagnostic dimensions of vulnerability, with emotional regulation gaining significant attention (Sawrikar et al., 2022). Emotion regulation involves modulating emotions, particularly how and when they are expressed or experienced, in relation to individual goals (Saccaro et al., 2024). Although some ambiguity exists regarding ERS categorization, adaptive and maladaptive categories are generally accepted. Adaptive ERS includes acceptance and positive reappraisal, while maladaptive ERS encompasses rumination (Navas-Casado et al., 2023). Various research domains highlight the pivotal role of emotion regulation in the onset and persistence of psychiatric disorders (Beauchaine, 2015), making it an ideal transdiagnostic construct for early intervention.

Assessing ERS among teenagers reliably is challenging with traditional questionnaires due to subjectivity. To meet the need for a more objective tool, the Reactivity and Regulation Situation Task (RRST) has been developed (Carthy et al., 2010). This naturalistic task presents participants with hypothetical scenarios, asking them to report their emotional responses (first thought and action) verbally. Trained researchers later classify these responses following Gross’ process model of emotion regulation (Samson et al., 2015). Although the RRST is more ecological and objective than questionnaires, the coding procedure remains somewhat subjective and semi-qualitative. This task has been used with anxious teens aged 10–17 years (Carthy et al., 2010) and with autistic individuals aged 8–20 years (Samson et al., 2015), measuring cognitive reappraisal improvements compared to matched subjects from the general population. Both studies demonstrated that the RRST effectively measures ERS in their respective populations and tracks changes during interventions, finding significant increases in cognitive reappraisal during a single visit.

With rising interest in non-pharmacological interventions, mindfulness has emerged as a potentially cost-effective and non-invasive method for improving mental health across various dimensions. Mindfulness meditation, rooted in Eastern tradition, has been integrated into Western psychological interventions (Kołodziejska & Paliński, 2022). It is defined as “awareness that arises from paying attention, on purpose, in the present moment, with a non-judgmental attitude” (Kabat-Zinn, 2005). This trait exists naturally at varying levels and can be enhanced through practice (Quaglia et al., 2016). Mindfulness-based interventions (MBI) have shown efficacy among adults for numerous psychosocial conditions, such as depression, anxiety, and stress (Zhang et al., 2021). For adolescents, outcomes for anxiety and attention are promising, though results remain inconclusive (Dunning et al., 2019; Galla et al., 2024; Johnson et al., 2024; Odgers et al., 2020). Contradictory evidence exists regarding MBIs’ effectiveness compared to active controls, their benefits for specific subgroups, and the underlying mechanisms (Alsubaie et al., 2017; Kuyken et al., 2022).

Given that adolescent mental health is a public health priority (McGorry et al., 2024), elucidating the clinical relevance of MBI as early interventions in non-clinical adolescents is a crucial research question that remains unanswered. This study aims to measure the impact of an MBI on ERS use in a representative sample of adolescents from the general population. We hypothesize that MBI increases the use of adaptive emotion regulation strategies, such as relaxation, cognitive reappraisal, and acceptance, while reducing action-related strategies like problem-solving.

## Method

### Participants and design

This study was a randomized controlled trial using an MBI to target both neurobiological outcomes and clinical outcomes, exploring stress, coping, and emotion regulation. The study recruited 70 non-clinical adolescents between 13 and 15 years old. Exclusion criteria were any psychiatric conditions (with the exception of current anxiety disorders or past depressive episodes), current psychotherapy or other co-intervention, claustrophobia, pregnancy, and/or inability to participate in groups. After inclusion, participants were stratified according to their score on the State-Trait Anxiety Inventory For Children (STAI-C) into a low anxiety group (≤31) or a high anxiety group (>31), based on a non-published median in a similar community sample. After stratification and one dropout, participants were randomized to either an Early (*N* = 33) or Late (*N* = 36) intervention group.

The Early group participated in three measurement visits, V0, V1, and V2, with baseline measurements split across V0 and V1, and the MBI taking place between V1 and V2. The Late group participated in four measurement visits, V0, V1, V2, and V2b, with their intervention taking place between V2 and V2b. When combining V0 and V1 as a single baseline visit, the main intervention effect was captured by the 2 × 2 design of Group × Visit, contrasting the change from V0/V1 to V2 between the two intervention groups, with the Late group acting as a passive control against the Early group, excluding an effect of mere time passing.

The final sample consisted of 40 girls and 29 boys, with mean baseline age 14.3 years old (range 13.1–16.0). A full overview of important baseline descriptives for the sample is presented in Table 1. The two intervention groups did not differ significantly at baseline in terms of age, gender distribution, as well as several biological, psychological, and intervention-related variables (e.g., puberty, anxiety disorder, participation duration).

**Table 1.** Baseline characteristics of the participant sample and intervention group comparisons. MBI: mindfulness based intervention, STAIT: state-trait anxiety inventory (LA: low anxiety, HA: high anxiety KSADS: kiddie schedule for affective disorders and schizophrenia).

Variable	Mean (SE)			ANOVA		
	All (69)	Early (33)	Late (36)	*F* / χ^2^	DF	*p*-val
V0 age (years)	14.3 (0.1)	14.3 (0.2)	14.3 (0.2)	0.00	(1.67)	0.9725
Duration total (days)	191.3 (9.7)	123.9 (6.5)	251.2 (9.6)	114.87	(1.66)	<0.0001
Duration V0–V2 (days)	128.2 (4.8)	123.9 (6.5)	132.0 (7.1)	0.71	(1.66)	0.4037
MBI sessions (out of 8)	7.5 (0.1)	7.5 (0.2)	7.5 (0.1)	0.01	(1.67)	0.9392
Gender (F/M)	40/29	22/11	18/18	1.34	(1)	0.2473
Puberty (No/Yes)	20/47	7/23	13/23	0.73	(1)	0.3921
Difficulties at V0 (No/Yes)	56/13	29/4	27/9	1.12	(1)	0.2899
Panic attacks (No/Yes)	59/10	26/7	33/3	1.38	(1)	0.2397
Social phobia (No/Yes)	62/7	28/5	34/2	0.85	(1)	0.3577
Anxiety disorder (No/Yes)	60/9	27/6	33/3	0.73	(1)	0.3922
STAIT strata (LA/HA)	20/49	8/25	12/24	0.32	(1)	0.5715

The protocol was approved by the Regional Research Ethical Committee on January 9th 2019. All participants and their legal representative signed the informed consent forms before the first visit. Participants were reimbursed for each research visit (i.e., from 15 to 62 US$) but not for their participation to group meditation intervention.

### Mindfulness-based intervention (MBI)

The intervention consisted of an 8-week MBI training, followed by mandatory 4 weeks of weekly booster sessions. Each weekly group session of up to 8 participants lasted 90 min. Participants were also encouraged to practice individually every day with the help of a smartphone app. The proposed MBI was an in-house adaptation from different mindfulness-based protocols, including MBCT (Mindfulness Based Cognitive Therapy) and MBSR (Mindfulness Based Stress Reduction), specially designed for young adolescents by trained mindfulness teachers and clinicians. Detailed description of the intervention can be found in the protocol. After completion of the intervention all participants retained access to the smartphone app, supporting a possible transition to a sustained practice.

The program was intended as in person groups of 8–12 adolescents with two trained instructors. However, due to the COVID pandemic, 1 out of the 8 groups had to use videoconferencing, and this has been adjusted for in the analyses.

### Reactivity and regulation situation task (RRST)

The RRST focuses on the use of spontaneous emotion regulation strategies through a behavioral task. The proposed task is adapted from Samson and colleagues (Carthy et al., 2010; Samson et al., 2015) and targets in a naturalistic way the evaluation of emotion regulation strategies used by the participants. This behavioral task lasts 15 min and uses hypothetical social and non-social scenarios (see Table 2). The aim is to study the spontaneous use of emotion regulation strategies, as well as prompting adaptive emotion regulation during a second exposure to the scenarios. For each scenario, participants write their first thought (free text), their stress level (likert scale from 1: none at all to 5: a lot) and the action they would take in this hypothetical situation (free text). The RRST is performed during the visits (either V0, V1, V2 or V2b). Early group participants performed the task twice: once at V0 or V1, once after the mindfulness training, at V2. Late group participants performed the task three times: once at V0 or V1, once at V2, then once more after the mindfulness training at V2b.

**Table 2.** The twelve hypothetical scenarios presented to the participants for the reactivity and regulation situation task (RRST).

Situation
1. Your mother tells you she needs to go to the doctor for a checkup.
2. You are walking down the street and a stranger approaches you.
3. You see several of your classmates hanging out together and you want to join them. When you do, you hear them laughing.
4. Your teacher asks to see you after class.
5. You enter a store and the employee stares at you.
6. Your father unexpectedly tells you that he will have to travel out of the country tomorrow.
7. You hear a knock on the door and when you open it you see a person you don’t know.
8. Your teacher gives an exam and says your result was surprising.
9. Your parents tell you they want to talk to you about something important.
10. You get presented an exam and are starting to read the questions.
11. You wake up in the middle of the night and head a loud noise in the hallway.
12. You are about to go out somewhere with a lot of people you just met.

### Data analysis

Data analysis proceeded in three steps, (a) ERS coding and rater agreement analysis, (b) descriptive analysis and (c) inferential modelling of ER choice.

#### Strategy coding and rater agreement

The written responses to the RRST task were coded according to a taxonomy of 16 ERS, which was derived from theoretical and empirical studies on ER classification (Allen & Windsor, 2019; Carthy et al., 2010; Goubet & Chrysikou, 2019; Gross, 1998, 2014; Koole, 2010; Naragon-Gainey et al., 2017). The full procedure involving coding, calculation of inter-rater agreement, and re-coding is presented in the Supplementary Material (Section S.1, and Table S1). At the end, 8 usable ERS remained for analysis: acceptance, behavioral disengagement, distraction, help seeking, problem solving, reappraisal, relaxation, and thought suppression. One participant was dropped from data analysis, due to their choices in the task not corresponding to any of the final 8, bringing the total participant sample for analysis to 68.

#### Descriptive analysis

For descriptive analysis, we first described the participant sample in terms of relevant baseline characteristics (see Table 1). Then, we calculated mean stress values and tabulated frequencies of ERS across groups (Early or Late) and visits (V0, V2, V2b), aggregated across RRST scenarios. As well, bivariate correlations between ERS were correlated to check if and how ERS were used together.

#### Inferential modelling

For inferential modelling, we analyzed how the MBI influenced ER choice. The primary analysis focused only on visits V0 and V2, to capture the main intervention effect. First, we formatted the data in long format, consisting of 2 × 12 × 8 rows per participants, corresponding to 2 visits (V0, V2), 12 RRST scenarios, and 8 coded ERS. Per row, there was a binary outcome coding whether or not the ERS was chosen for that particular scenario and visit. These data were entered into a multilevel logistic regression (a.k.a., generalized linear mixed model, or GLMM), modelling the probability of choosing a regulation strategy or not, depending on a four-way design of Group × Visit × Strategy × Stress. The Group × Visit part of the design reflected the intervention effect, in that change in regulation between V0 and V2 was only expected for the Early group. However, this intervention effect was expected to be strongly dependent on the type of ERS used (Group × Visit × Strategy), such that, e.g., the probability of using of relaxation may increase for the Early group after the intervention, whereas no such change may occur for problem solving. Finally, the level of stress was included as a moderator (Group × Visit × Strategy × Stress), to allow that changes in ER choice due to the intervention depended on stress.

Once the GLMM model was fitted, a Type II analysis of deviance was calculated for the design effects with Wald chi-square tests. For a significant Group × Visit × Strategy interaction, we conducted follow-up chi-square tests of Group × Visit within levels of ERS. Significant interactions among these were followed up with pairwise visit contrasts within intervention groups, using Wald *z*-tests. In the event a significant Group × Visit × Strategy was found, follow-up modelling was planned that included the V2b measurement for the Late group. For this analysis, the Visit variable would be recoded, such that V0 and V2 became “Pre” and “Post” for the Early group, and V2 and V2b became “Pre” and “Post” for the Late group. As such, subsequent modelling would allow estimating the pooled intervention effect across both groups, potentially gaining power. However, this analysis was conditioned on a significant Group × Visit × Strategy effect for the V0-V2 data, since otherwise the analysis cannot exclude the possibility of an effect due to mere time passing.

Further technical details regarding multilevel modelling–including rationale for the use of a GLMM—is elaborated in the Supplementary Material (Section S.2).

### Software

All analyses were conducted using the R statistical software, version, with packages lme4 and car for GLMM modelling, and package emmeans for follow-up tests (Bates et al., 2015; Fox & Weisberg, 2019; Lenth, 2022).

## Results

### Descriptive analysis

Results of the descriptive analysis are presented in Table 3. Of 1240 coded cases (with a self-reported stress level larger than 1), problem solving was the most popular strategy (29.0%), followed by reappraisal (20.5%). No regulation was used in only 5.8% of cases, although this was still larger than the use of distraction and thought suppression (or the ERS that were eliminated during ER coding). More than one ERS was used in 11.9% of cases, with the maximum amount of simultaneous strategies 3 (0.2%). Correlations between the use of ERS were generally small (*r* < |0.20|), suggesting that the use of more than one ERS simultaneously was relatively rare. The strongest correlation was found between the use of distraction and thought suppression, *r* = 0.33. Use of problem solving had a weak negative correlation with the use of relaxation, acceptance, and reappraisal (see Supplemental Material Figure S1 for the full correlation matrix).

**Table 3.** Mean/SD stress level and frequency of ERS use by group and by visit.

ERS	Early		Late			Total
	V0	V2	V0	V2	V2b	
Acceptance	34	29	23	45	30	161
Behavioral disengagement	36	25	23	17	20	121
Distraction	10	5	10	5	5	35
Help seeking	49	50	51	41	47	238
Problem solving	83	83	62	79	53	360
Reappraisal	43	52	56	46	57	254
Relaxation	17	33	19	10	44	123
Thought suppression	6	1	6	1	2	16
No regulation	18	11	12	10	19	72
Multiple ERS	40	40	28	21	17	146
Stress	2.48 (1.2)	2.39 (1.2)	2.22 (1.2)	2.04 (1.1)	2.10 (1.1)	

*Note.* ERS = emotion regulation strategy.

### Inferential modelling of ERS use

**Figure 1.**
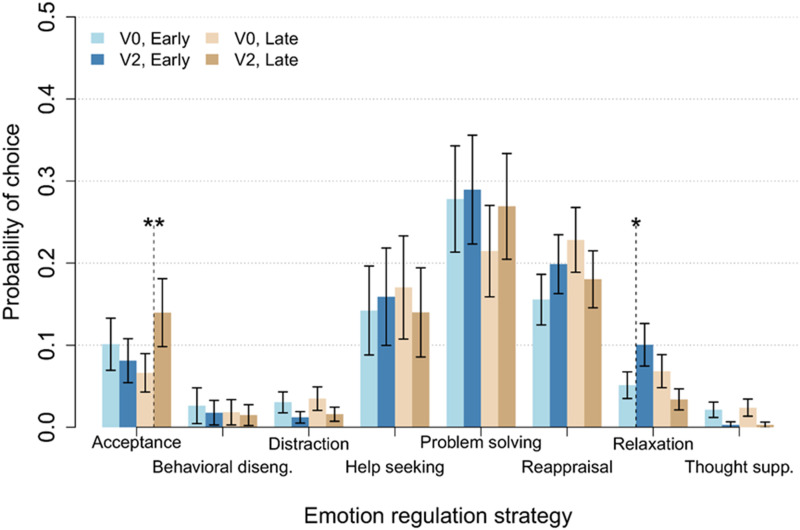
Model-based (GLMM) estimates of ERS use probabilities and standard errors per group, visit, and emotion regulation strategy. GLMM: generalized linear mixed model. ERS: emotion regulation strategy.

A multilevel logistic regression (GLMM) was fitted to the ER choice data, with a design of Group × Visit × Strategy × Stress. Non-significant interactions involving stress were first removed to reduce model complexity. This included the four-way interaction, which suggested that stress level did not modify the intervention effect on specific strategies, χ^2^(10) = 7.97, *p* = .6317. The full Type II analysis of deviance breakdown of effects is listed in Table 4. The three-way interaction of Group × Visit × Strategy was significant, χ^2^(7) = 19.18, *p* = .0076, indicating that change in regulation use differed between the two intervention groups, but was specific to certain ERS. Follow-up chi-square tests of the intervention effect within ERS revealed significant Group × Visit interactions only for acceptance, χ^2^(1) = 6.78, *p* = .0092, and relaxation, χ^2^(1) = 7.66, *p* = .0057. Follow-up *z*-tests of the visit effect within groups indicated that, for relaxation, there was a significant increase in the use of relaxation for the Early group, *z* = −2.12, *p* = .0340, but not for the Late group, *z* = 1.76, *p* = .0786. For acceptance, contrary to expectations, there was a significant increase in the use of acceptance in the Late group, *z* = −2.75, *p* = .0059, but not the Early group, *z* = 0.84, *p* = .3995. A graphical summary of Group × Visit × Strategy probabilities is presented in Figure 1.

**Table 4.** Type II analysis of deviance of the multilevel logistic regression GLMM for the main intervention effect.

Effect	χ2	DF	*p*-value
Group	0.28	1	.5971
Visit	0.07	1	.7887
Strategy	126.83	7	<.0001***
Stress	1.37	1	.2413
Group × visit	0.30	1	.5851
Group × strategy	7.05	7	.4238
Visit × strategy	11.97	7	.1017
Visit × stress	1.29	1	.2565
Strategy × stress	46.24	7	<.0001***
Group × visit × strategy	19.21	7	.0075**
Visit × strategy × stress	12.80	7	.0771

*p*-value coding: 0 ‘***’ .001 ‘**’ .01 ‘*’ .05.

The full four-way model contained a significant interaction of Visit × Strategy × Stress, which was no longer significant in the reduced model, hence we did not interpret this interaction further. There was a significant Strategy × Stress interaction, however, χ^2^(7) = 46.24, *p* < .0001, indicating that, regardless of the intervention, ERS preference shifted as a function of stress values. Follow-up z-tests for stress slopes within ERS revealed that there were significant slopes for acceptance, β = −0.44, *z* = −3.50, *p* = .0005, behavioral disengagement, β = 0.59, *z* = 4.04, *p* = .0001, and relaxation, β = 0.47, *z* = 3.63, *p* = .0003. Specifically, although problem solving was the preferred ERS across all stress levels, the probability of using acceptance decreased with stress, from being the third preferred strategy at stress level 2, to being only the seventh preferred strategy at stress level 5. The probability of both behavioral disengagement and relaxation use increased with stress level, though less dramatically so than acceptance use decreased. A full list of all effects for the main intervention data can be found in the Supplementary Material, Section S.4, including Group × Visit effects within strategies, pairwise Visit contrasts within groups and strategies, and Stress slopes within visits and strategies (Tables S2–4).

### Pooled pre-post modelling

**Figure 2.**
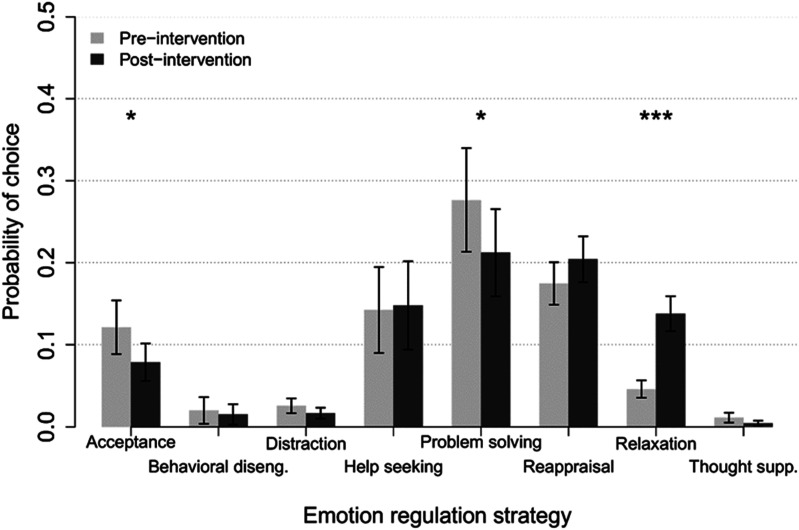
Model-based (GLMM) estimates of ERS use probabilities and standard errors for pre-post pooled intervention groups, per emotion regulation strategy. GLMM: generalized linear mixed model. ERS: emotion regulation strategy.

**Figure 3.**
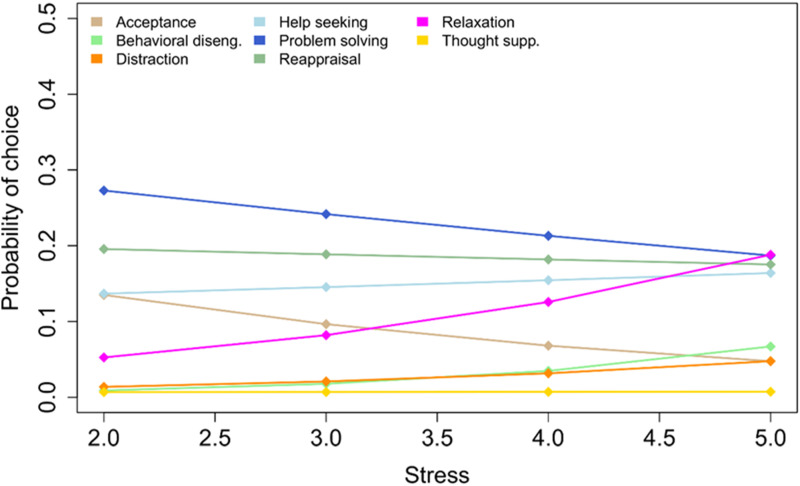
GLMM estimates of ERS use probabilities for increasing stress levels for pooled pre-post intervention data. GLMM: generalized linear mixed model. ERS: emotion regulation strategy.

Because the main ER choice analysis established the Group × Visit × Strategy intervention effect, we were allowed to pool the two intervention groups and analyze the Late group’s intervention effect by involving the V2b visit. Visit times were recoded to pre- and post-time (V0-V2 for Early; V2-V2b for Late), and the data resubmitted to the GLMM in a Time × Strategy × Stress model. The initial result indicated that there was no significant three-way interaction, nor a significant Time × Stress interaction, hence these effects were removed from the final model. The final model contained a highly significant Time × Strategy interaction, χ^2^(7) = 41.67, *p* < .0001. Follow-up *z*-tests indicated that there was significant decrease in use of acceptance, *z* = 2.51, *p* = .0119, significant decrease in use of problem solving, *z* = 2.31 *p* = .0208, and highly significant increase in the use of relaxation, *z* = −5.06, *p* < .0001. A graphical summary of Time × Strategy probabilities is presented in Figure 2. The final model also contained a highly significant Strategy × Stress interaction, χ^2^(7) = 55.03, *p* < .0001. Follow-up *z*-tests on stress slopes per ERS indicated that the pattern of results remained identical to the model without pooled intervention groups, with significant stress slopes for acceptance, behavioral disengagement, and relaxation. However, the relaxation effect was more pronounced than for the unpooled data, with relaxation overtaking problem solving as the preferred strategy at the highest stress level (see Figure 3).

A full list of all effects for the main intervention data can be found in the Supplementary Material, Section S.5, including the Time × Strategy × Stress ANOVA breakdown, pairwise Time contrasts within strategies, and Stress slopes within strategies (Tables S5–7).

## Discussion

The present study aimed to investigate the impact of a MBI on emotion regulation (ER) strategy use in adolescents from the general population. Our findings reveal that the MBI significantly influenced the use of specific ERS, notably increasing the use of relaxation while decreasing the use of acceptance and problem-solving strategies. We also found that acceptance, behavioral disengagement and relaxation vary with the stress level, relaxation being the preferred strategy for a higher stress level. These results provide insights into the potential mechanisms through which MBI may benefit mental health in adolescents, particularly by enhancing specific adaptive ERS.

Our study demonstrates that adolescents who participated in the MBI showed a significant increase in the use of relaxation. This is consistent with the core principles of mindfulness, which emphasize present-moment awareness and mind-body connection. This effect is important because relaxation is shown to be a protective factor against stress (Rogerson et al., 2024). Mindfulness shares common exercises with relaxation, such as respiration techniques, or body scan, however, it also affects cognitive processes and is expected to improve cognitive reappraisal. Since we only found an effect on relaxation, our interpretation is that in such a short time frame, we tend to observe only the relaxation effect of mindfulness, one of the first component to be integrated by participants, and not the more complex strategies, such as cognitive reappraisal (Luberto et al., 2020). Conversely, we observed a decrease in the use of acceptance and problem-solving strategies among adolescents who underwent the MBI. The reduction in acceptance may seem counterintuitive, given that mindfulness encourages acceptance of thoughts and feelings(Lindsay & Creswell, 2017). A possible explanation for this might be the main challenge during coding arose when acceptance was coded for high stress levels. Indeed, identifying true acceptance, an adaptive ERS (“I don’t do anything” in the sense that I accept the situation as it is), and distinguishing it from freezing, a maladaptive ERS (“I don’t do anything” in the sense that I am not able to think of moving) is unreliable given the short sentence recorded. This may lead to an overestimate of acceptance in the results. Another hypothesis that could support this result is that of the amygdala highjack (Ressler, 2010). This hypothesis states that under low levels of stress, the prefrontal cortex functions normally, so elaborate cognitive ERS such as acceptance levels are high; whereas under high levels of stress, the prefrontal cortex is inhibited by amygdala activity, leading to the use of less complex reactions such as freezing. This would explain why participants report lower levels of acceptance at high stress levels. Concerning problem solving, it is unsurprisingly the most used ERS, however its decrease after the intervention may indicate that it was replaced with other strategies, less oriented on situation modification.

During adolescence new challenges arise, such as increasing independence from caregivers, who shape ER in childhood, and navigating significant changes such as social and biological. Many individuals respond by improving their ER skill via the maturation of affective control (Silvers, 2022). However, some are overwhelmed and this can lead to emotional dysregulation, a risk factor for psychopathology. The aim of this study was to improve this learning curve, however, 13–15 years old might still be too young for participants to fully appreciate all the components of mindfulness. Indeed, recent research done in schools advocates for intervention either before this developmental stage, or later (Johnson et al., 2024; Yeager et al., 2018).

Nevertheless, our findings of more frequent use of relaxation after MBI, especially for situation generating a high level of stress, does confirm the positive impact of MBI on stress management. One explanation may be that after the MBI, participants discriminate bodily sensations better. This would help realize that when stressed, the best strategy is first to relax, because acceptance for high stress levels would actually involve passivity and giving up.

Comparing our results with the previous studies using the RRST is somewhat limited, since they involved only one visit focused on introducing appraisal, whereas ours has two or three visits and is focused solely on MBI. The first study focuses solely on inducing the use of cognitive reappraisal and does not report the use of other ERS (Carthy et al., 2010). The second one shows that the two most prominent ERS was problem solving (up to 50%) and reappraisal, which is similar to our baseline results (Samson et al., 2015).

It should be noted that the study’s primary outcomes were neuroimaging and self-reported questionnaires, such as STAI-C and Beck’s depression inventory (BDI). When comparing the two groups, the main study shows no main effect when analyzing the neuroimaging or questionnaires (Piguet et al., 2025). Thus, this study’s semi-qualitative finding is a promising lead on understanding the mechanism of action through which mindfulness helps improve mental health among our population.

### Strengths and limitations

A key strength of our study is the robust experimental design, with a control group excluding effects due to mere time passing and randomized condition assignment excluding baseline confounding. Additionally, the strict recruiting criteria ensured a homogeneous sample, enhancing the internal validity of our findings. The ecological nature of the RRST is thought to be more robust than a questionnaire. However, the main limitation of this study is that there is still an inherent subjective part to the RRST, both by the participants and the coders. What we call acceptance in this article is more likely closer to resignation, unaffected, or no regulation. To minimize this, we used convergent measures and checked interrater reliability to ensure consistency across assessments.

### Conclusions and perspectives

To conclude, MBI helps adolescents from the general population, aged 13 to 15, to use relaxation when their stress level is high. These results shed light on the positive impact of mindfulness on ERS use, through relaxation. This brings further evidence that ERS can be taught with 8-week MBI protocols. This study is a promising lead in identifying the specific mechanisms of MBI on ERS use among adolescents. We did not find any change in cognitive reappraisal use, which corresponds to recent findings among 13–15 years old. Such findings could prove useful for implementing wider-scale research and developing targeted interventions. The ultimate goal is to design cost-efficient, non-invasive, and non-pharmacological interventions to limit the increasing socioeconomic burden of psychiatric disorders, especially concerning youth mental health, which is a public health priority. Moreover, exploring the long-term effects of MBI on emotional and psychological well-being could provide more comprehensive insights into its therapeutic potential and its target audience.

## Supplemental Material

Supplemental Material - Mindfulness shapes emotion regulation in non-clinical adolescents: Secondary outcomes of a randomized controlled trial Supplemental Material for Mindfulness shapes emotion regulation in non-clinical adolescents: Secondary outcomes of a randomized controlled trial by Erik Mendola, Ben Meuleman, Mariana Magnus Smith, Vladimira Ivanova, Eléonore Pham, Valentine Savary, Patricia Cernadas, Zeynep Celen, Luigi Francesco Saccaro, Andrea Samson, Paul Klauser, Arnaud Merglen, Camille Marie Piguet in Clinical Child Psychology and Psychiatry.
