# Supplementary material for: Mindfulness shapes emotion regulation in non-clinical adolescents: Secondary outcomes of a randomized controlled trial
Source: Clin Child Psychol Psychiatry. 2025 Sep 24;31(1):195–211. doi: 10.1177/13591045251377898 (PMC12705890; doi:10.1177/13591045251377898)
Supplement: Supplemental Material - Mindfulness shapes emotion regulation in non-clinical adolescents: Secondary outcomes of a randomized controlled trial [file sj-pdf-1-ccp-10.1177_13591045251377898.pdf]

# Mindfulness Enhances Emotion Regulation Strategies in Healthy Adolescents: Secondary Outcomes of a Randomized Controlled Trial

# Supplementary Material

The following is supplementary material to the main results presented in Mendola et al. (2024), relating to the impact of a randomized controlled mindfulness intervention on the use of emotion regulation strategies. This supplementary material includes **(1)** details regarding the regulation coding of participants' written responses, as well as rater agreement among coders, **(2)** rationale and details on multilevel modelling, **(3)** descriptive correlations between regulation strategies, and **(4)** follow-up tests and pairwise contrasts for unpooled data, and **(5)** follow-up tests and pairwise contrasts for pooled data.

## S.1 Regulation strategy coding and rater agreement

The written responses to the RRST task were coded according to a taxonomy of 16 ERS, which was derived from theoretical and empirical studies on ER classification (Allen et Windsor 2019; Carthy et al. 2010; Goubet et Chrysikou 2019; Gross 1998; 2014; Koole 2010; Naragon-Gainey, McMahon, et Chacko 2017). Two independent raters performed this coding, allowing more than one ERS per written response (i.e., non-exclusive coding). This approach was favored over exclusive coding, due to reflecting real-life use of multiple strategies as well as simplifying statistical analyses (see further). For the RRST task, a written response for a scenario was only prompted if the participant rated their level of stress for the scenario larger than 1. This amounted to 1347 cases to be coded, a further 107 of which had a missing written response, bringing the total amount of cases to be coded to 1240. When the rater judged an ERS to be present, it was coded as 1, and 0 when absent. When the rater judged that no regulation had been indicated by the written response, all ERS were coded 0 for that case.

After the initial coding, 8 of 16 ERS were removed for subsequent analyses due to occurring less than 30 times (and sometimes not at all). This included avoidance, rumination, humour, catastrophizing, emotionally expressive coping, expressive suppression, exercising, and substance use. The remaining 8 strategies were each submitted to an analysis of rater agreement, using McNemar's test for disagreement in 2×2 paired classification tables, and Cohen's Kappa as a measure of rater agreement strength. Results of the reliability analysis are presented in Table S1. Only for three ERS we found satisfactory rater agreement, for behavioral disengagement, help seeking, and relaxation, each having strong to perfect Kappa values and a non-significant McNemar test. For the remaining five strategies, Kappa values were moderate to poor, with highly significant McNemar tests. To solve disagreements, a third independent coder decided all 634 disagreement cases, by agreeing with one of the other two raters (no new codings were permitted). In this way, a final set of ER codings was obtained for analysis.

**Table S1.** Rater agreement for the initial ER coding among two independent raters based on McNemar's test for paired categorical data. Qualitative interpretation of Cohen's Kappa (McHugh 2012). †: Note that the total number of codings is larger than the number of cases, due to each case allowing more than one ERS to be present. *P*-value coding: 0 '***' 0.001 '**' 0.01 '*' 0.05

| Strategy | Agree | Disagree | Total | Kappa | McHugh | *p*-value | |
|---|---|---|---|---|---|---|---|
| *Acceptance* | 54.2% | 45.8% | 236 | 0.653 | Moderate | 0.0000 | *** |
| *Behavioral disengagement* | 75.0% | 25.0% | 136 | 0.842 | Strong | 1.0000 | |
| *Distraction* | 55.0% | 45.0% | 40 | 0.703 | Moderate | 0.0004 | *** |
| *Help seeking* | 85.4% | 14.6% | 253 | 0.903 | Perfect | 0.7423 | |
| *Problem solving* | 54.7% | 45.3% | 552 | 0.568 | Weak | 0.0000 | *** |
| *Reappraisal* | 59.9% | 40.2% | 259 | 0.702 | Moderate | 0.0000 | *** |
| *Relaxation* | 85.4% | 14.6% | 137 | 0.912 | Perfect | 1.0000 | |
| *Thought suppression* | 5.6% | 94.4% | 71 | 0.086 | None | 0.0000 | *** |
| **Total†** | 62.1% | 37.9% | 1684 | | | | |

## S.2 Rationale and details on multilevel modelling

For inferential modelling, we analyzed how the MBI influenced ER choice. The primary analysis focused only on visits V0 and V2, to capture the main intervention effect. First, we formatted the data in long format, consisting of 2 × 12 × 8 rows per participant, corresponding to 2 visits (V0, V2), 12 RRST scenarios, and 8 coded ERS. Per row, there was a binary outcome coding whether or not the ERS was chosen for that particular scenario and visit. These data were entered into a multilevel logistic regression (a.k.a., generalized linear mixed model, or GLMM), modelling the probability of choosing to regulate or not, depending on a four-way design of Group × Visit × Strategy × Stress. The Group × Visit part of the design reflected the intervention effect, in that change in regulation between V0 and V2 was only expected for the Early group. However, this intervention effect was expected to be strongly dependent on the type of ERS used (Group × Visit × Strategy), such that, e.g., the probability of using of relaxation may increase for the Early group after the intervention, whereas no such change may occur for problem solving. Finally, the level of stress was included as a moderator (Group × Visit × Strategy × Stress), to allow that changes in ER choice due to the intervention depended on Stress.

Multilevel models such as a GLMM are appropriate for data with hierarchical levels of clustering, which in our case consisted of having ER choices measured repeatedly within the 12 scenarios and 69 participants. Participants and scenarios are typically considered as random sources of measurement error, in that different studies will sample different subsets from the population of participants and scenarios (Fitzmaurice, Laird, et Ware 2004). To account for correlation of choices within these two levels, and treating them as sources of random error, the model included two separate random intercepts for scenarios and participants. In addition, exploratory modelling optimizing Akaike's Information Criterion (AIC) showed evidence that there were differences in ER choice bias between the 12 scenarios, but not between participants. Therefore, the analysis model included a random slope for ER choice within scenarios.

Once the GLMM model was fitted, a Type II analysis of deviance was calculated for the design effects with Wald chi-square tests. To reduce the complexity of the model, non-significant effects

involving stress were removed, following which the model was refitted and the analysis of deviance was repeated. For a significant Group × Visit × Strategy interaction, we conducted follow-up chi-square tests of Group × Visit within levels of ERS. Significant interactions among these were followed up with pairwise visit contrasts within intervention groups, using Wald *z*-tests. For significant interactions that involved the stress moderator, follow-up analyses consisted of Wald *z*-tests on stress slopes for the relevant conditions, to investigate how the probability of ER choice increased or decreased for changing stress values. For all Type II analyses of deviance, we ignored effects that were conceptually uninterpretable, such as Group effects without Visit effect, Visit effects without Group effect, and any effect not involving ERS. The latter is due to the fact that the choice to regulate otherwise has no interpretable meaning when collapsed across specific ERS.

In the event a significant Group × Visit × Strategy was found, follow-up modelling was planned that included the V2b measurement for the Late group. For this analysis, the Visit variable would be recoded, such that V0 and V2 became “Pre” and “Post” for the Early group, and V2 and V2b became “Pre” and “Post” for the Late group. As such, subsequent modelling would allow estimating the pooled intervention effect across both groups, potentially gaining power. However, this analysis was conditioned on a significant Group × Visit × Strategy effect for the V0-V2 data, since otherwise the analysis cannot exclude the possibility of an effect due to mere time passing.

## S.3 Descriptive correlations between regulation strategies

Correlations between the use of different regulation strategies was calculated by first decorrelating strategy use variables for repeated measures between visits, within participants, and within scenarios, using the residual data of a multilevel regression controlling for these sources of repeated measures correlation. Next, bivariate Pearson correlations were calculated on the decorrelated data. Figure S1 depicts these correlations, with values below |0.20| suppressed as being practically unimportant. As well, the ERS were reordered based on their correlational similarity with hierarchical clustering, to reveal interesting groupings—if any. In general, the correlations were small, owing to the fact that the use of more than one ERS simultaneously was relatively rare. The strongest correlation was found between the use of distraction and thought suppression, $r = 0.33$. Use of problem solving had a weak negative correlation with the use of relaxation, acceptance, and reappraisal.

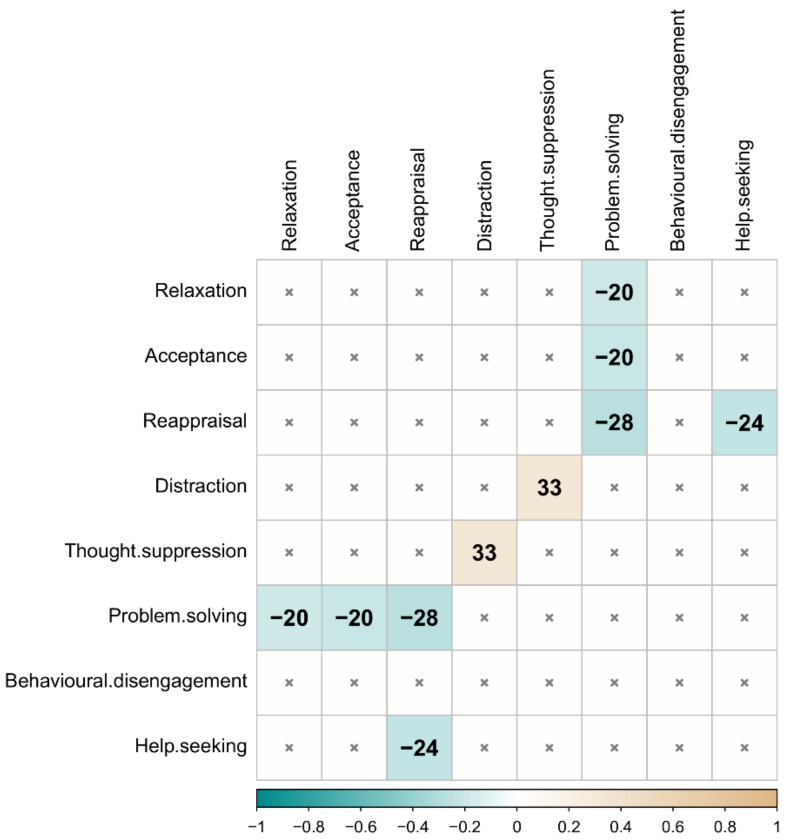


**Figure S1.** Bivariate Pearson correlations among emotion regulation strategies, converted to 0–100 scale. Correlations below |0.20| were suppressed for clarity, and strategies reordered according to their correlational similarity.

## S.4 Follow-up tests and pairwise contrasts for unpooled data

The following tables contain follow-up tests and pairwise contrasts for the main intervention data, that is, unpooled across intervention groups. This includes Group × Visit interaction tests within regulation strategies (Table S2), pairwise visit contrasts within groups and regulation strategies (Table S3), and stress slopes within visits and regulation strategies (Table S4).

**Table S2.** Group × Visit interaction tests within regulation strategies (likelihood ratio tests). *P*-value coding: 0 '***' 0.001 '**' 0.01 '*' 0.05

| **Regulation strategy** | $\chi^2$ | **DF** | ***p*-value** | |
|---|---|---|---|---|
| *Acceptance* | 6.78 | 1 | 0.0092 | ** |
| *Behavioral disengagement* | 0.14 | 1 | 0.3246 | |
| *Distraction* | 0.03 | 1 | 0.8626 | |
| *Help seeking* | 0.97 | 1 | 0.3246 | |
| *Problem solving* | 0.62 | 1 | 0.4306 | |
| *Reappraisal* | 3.27 | 1 | 0.0706 | * |
| *Relaxation* | 7.66 | 1 | 0.0057 | ** |

| | | | |
|---|---|---|---|
| *Thought suppression* | 0.02 | 1 | 0.9010 |

**Table S3.** Pairwise visit contrasts within groups and regulation strategies (*z*-tests). OR: odds ratio; SE: standard error. *P*-value coding: 0 ‘***’ 0.001 ‘**’ 0.01 ‘*’ 0.05.

| Strategy | Contrast | OR | SE | *z*-value | *p*-value | |
|---|---|---|---|---|---|---|
| *Acceptance* | Early: V0–V2 | 1.28 | 0.37 | 0.85 | 0.3958 | |
| | Late: V0–V2 | 0.44 | 0.13 | -2.75 | 0.0059 | ** |
| *Behavioral disengagement* | Early: V0–V2 | 1.50 | 0.50 | 1.22 | 0.2210 | |
| | Late: V0–V2 | 1.24 | 0.47 | 0.57 | 0.5718 | |
| *Distraction* | Early: V0–V2 | 2.58 | 1.58 | 1.55 | 0.1215 | |
| | Late: V0–V2 | 2.24 | 1.29 | 1.40 | 0.1624 | |
| *Help seeking* | Early: V0–V2 | 0.88 | 0.22 | -0.52 | 0.6058 | |
| | Late: V0–V2 | 1.26 | 0.34 | 0.87 | 0.3860 | |
| *Problem solving* | Early: V0–V2 | 0.95 | 0.20 | -0.27 | 0.7877 | |
| | Late: V0–V2 | 0.74 | 0.17 | -1.34 | 0.1817 | |
| *Reappraisal* | Early: V0–V2 | 0.74 | 0.17 | -1.27 | 0.2026 | |
| | Late: V0–V2 | 1.35 | 0.31 | 1.28 | 0.2000 | |
| *Relaxation* | Early: V0–V2 | 0.48 | 0.17 | -2.12 | 0.0338 | * |
| | Late: V0–V2 | 2.08 | 0.87 | 1.76 | 0.0785 | |
| *Thought suppression* | Early: V0–V2 | 7.01 | 8.30 | 1.64 | 0.1001 | |
| | Late: V0–V2 | 8.41 | 10.39 | 1.72 | 0.0848 | |

**Table S4.** Stress slopes per visit per strategy on a log-odds scale (*z*-tests). SE: standard error. *P*-value coding: 0 ‘***’ 0.001 ‘**’ 0.01 ‘*’ 0.05.

| Strategy | Visit | β | SE | *z*-value | *p*-value | |
|---|---|---|---|---|---|---|
| Acceptance | V0 | -0.59 | 0.18 | -3.20 | 0.0014 | ** |
| | V2 | -0.29 | 0.16 | -1.77 | 0.0768 | |
| Behavioral disengagement | V0 | 0.62 | 0.19 | 3.18 | 0.0015 | ** |
| | V2 | 0.56 | 0.20 | 2.79 | 0.0053 | |
| Distraction | V0 | 0.10 | 0.26 | 0.40 | 0.6859 | |
| | V2 | 0.81 | 0.35 | 2.33 | 0.0200 | * |
| Help seeking | V0 | 0.24 | 0.14 | 1.71 | 0.0879 | |
| | V2 | -0.02 | 0.15 | -0.15 | 0.8800 | |
| Problem solving | V0 | 0.06 | 0.12 | 0.49 | 0.6253 | |
| | V2 | -0.32 | 0.12 | -2.61 | 0.0090 | ** |
| Reappraisal | V0 | 0.04 | 0.13 | 0.35 | 0.7232 | |
| | V2 | -0.15 | 0.13 | -1.12 | 0.2650 | |
| Relaxation | V0 | 0.32 | 0.19 | 1.71 | 0.0882 | |
| | V2 | 0.63 | 0.18 | 3.51 | 0.0004 | *** |
| Thought suppression | V0 | 0.06 | 0.32 | 0.19 | 0.8496 | |
| | V2 | -0.68 | 1.01 | -0.67 | 0.5018 | |

## S.5 Follow-up tests and pairwise contrasts for pooled data

The following tables contain follow-up tests and pairwise contrasts for data that was pooled across intervention to common pre-post intervention visits (V0-V2 for Early; V2-V2b for Late). This includes the Time × Strategy × Stress ANOVA breakdown for the full model (Table S5), pairwise Time contrasts within regulation strategies (Table S6), and stress slopes within regulation strategies (Table S7).

**Table S5.** Time × Strategy × Stress ANOVA breakdown for the pooled-data model (Wald chi-square tests). *P*-value coding: 0 '***' 0.001 '**' 0.01 '*' 0.05.

| Effect | $\chi^2$ | DF | *p*-value | |
|---|---|---|---|---|
| Time | 0.31 | 1 | 0.5786 | |
| Strategy | 84.17 | 7 | <0.0001 | *** |
| Stress | 1.67 | 1 | 0.1963 | |
| Time × Strategy | 42.22 | 7 | <0.0001 | *** |
| Time × Stress | 0.90 | 1 | 0.3441 | |
| Strategy × Stress | 55.81 | 7 | <0.0001 | *** |
| Time × Strategy × Stress | 3.19 | 7 | 0.8667 | |

**Table S6.** Pairwise Time contrasts within regulation strategies on an odds-ratio (OR) scale(*z*-tests). *P*-value coding: 0 '***' 0.001 '**' 0.01 '*' 0.05.

| Strategy | Contrast | OR | SE | *z*-value | *p*-value | |
|---|---|---|---|---|---|---|
| Acceptance | Pre–Post | 1.62 | 0.32 | 2.46 | 0.0139 | * |
| Behavioral disengagement | Pre–Post | 1.33 | 0.33 | 1.12 | 0.2617 | |
| Distraction | Pre–Post | 1.56 | 0.65 | 1.06 | 0.2882 | |
| Help seeking | Pre–Post | 0.96 | 0.18 | -0.24 | 0.8080 | |
| Problem solving | Pre–Post | 1.42 | 0.21 | 2.31 | 0.0210 | * |
| Reappraisal | Pre–Post | 0.82 | 0.13 | -1.19 | 0.2325 | |
| Relaxation | Pre–Post | 0.30 | 0.07 | -5.00 | <0.0001 | *** |
| Thought suppression | Pre–Post | 2.50 | 1.74 | 1.32 | 0.1877 | |

**Table S7.** Stress slopes within-regulation strategies on a log-odds scale (*z*-tests). SE: standard error. *P*-value coding: 0 '***' 0.001 '**' 0.01 '*' 0.05.

| Strategy | β | SE | *z*-value | *p*-value | |
|---|---|---|---|---|---|
| Acceptance | -0.39 | 0.12 | -3.22 | 0.0013 | ** |
| Behavioral disengagement | 0.69 | 0.15 | 4.63 | <0.0001 | *** |
| Distraction | 0.42 | 0.22 | 1.91 | 0.0568 | |

| | | | | | |
|---|---|---|---|---|---|
| Help seeking | 0.08 | 0.10 | 0.69 | 0.4887 | |
| Problem solving | -0.16 | 0.09 | -1.91 | 0.0568 | |
| Reappraisal | -0.04 | 0.09 | -0.46 | 0.6448 | |
| Relaxation | 0.49 | 0.12 | 4.23 | <0.0001 | *** |
| Thought suppression | 0.01 | 0.37 | 0.02 | 0.9808 | |

## S.6 Adaptation of the MBI-protocol

**Table S8.** Detailed sessions for the adapted MBI-protocol used for the study. MBI : Mindfulness-based intervention

| | Session Theme | Agenda | Home practise |
|---|---|---|---|
| 1 | ATTENTION and AUTOPILOT | | |
| | Introduction to attention and autopilot mode Beginners Mind | Group and instructor introduction Mindfulness definition: To pay attention to what happens in the present moment, with a curious and non-judgmental attitude. Dialogue about attention awareness and focus of attention The six channels: five senses and thoughts Practice: Eating a raisin as an explorer Practice: Grounding mediation Closure practice | Chart: Attention - where is my attention now? Practice: Mindful eating – a mindful bite once a day Practice: Grounding meditation |
| 2 | DISCOVERING THE BODY LANGUAGE | | |
| | Discovering bodily sensations Acceptance | Opening practice: Grounding meditation<br>Dialogue about home practice<br>Dialogue about sensations and sensation awareness<br>Practice: Lying down Body scan, with a component of contraction and relaxation at the beginning<br>Practice: Seated body scan<br>Closure practice | Chart: Cool moment of the day Practice: Doing mindfully something habitually done on autopilot Practice: Grounding meditation Practice: Body scan |
| 3 | ATTENTION STABILIZATION | | |
| | Discovering the breath Non-striving | Opening practice: Grounding meditation<br>Dialogue about home practice<br>Dialogue about the breath and it's use as a possible anchor<br>Practice: The 3 min break<br>Practice: Stop and breath<br>Practice: Siting practice focusing on breath<br>Closure practice | Practice: Body scan<br>Practice: 3 min break<br>Practice: Stop and breath |
| 4 | RECOGNIZING EMOTIONS | | |
| | Recognizing emotions from bodily | Opening practice: Grounding meditation Dialogue about home practice Practice: Seated body scan, including emotions Dialogue about emotions: | Chart: Emotions - recognizing links between sensations, |

| | | | |
|---|---|---|---|
| | sensations Patience | recognizing emotions from bodily sensations and the link between sensations, thoughts and behaviour. Practice: Sitting meditation including emotions (internal forecast) Drawing and naming the identified emotion Closure practice | thoughts and behaviour Practice: The 3 min break Practice: Sitting meditation including emotions |
| 5 | RECOGNIZING THOUGHTS | | |
| | I'm much more than my thoughts Non-judging | Opening practice: Grounding meditation Dialogue about home practice Walking down the street exercise Discussion about thoughts and emotions Practice: walking meditation Closure practice | Practice: Sitting meditation including emotions Practice: Walking mediation |
| 6 | AUTOMATIC REACTION OR CONSCIOUS RESPONSE? | | |
| | Exploring stressors and stress reaction Letting go | Opening practice: Grounding meditation Dialogue about home practice Practice: Open awareness Quick board game: identifying stress reaction Discussion about stressors and stress reaction strategies Practice: 5 min to deal with stress Closure practice | Chart: Identifying qualities on others Practice: 5 min to deal with stress Practice: Walking mediation Practice: Open awareness |
| 7 | KINDNESS | | |
| | Being kind to oneself and to others Gratitude and generosity | Opening practice: Grounding meditation Dialogue about home practice Discussion about kindness and compassion Exercise about our own qualities Practice: finding refuge Drawing our refuge Mindful listening exercise Dialogue about communications and social media Closure practice | Letter to oneself: What did I learned and I do not want to forget? What did I learn about myself? Which meditations do I want to keep practicing? Practice: Refuge Practice: Chose another meditation to practice |
| 8 | CLOSURE AND OPENNESS | | |
| | Integrating the program Trust | Opening practice: Grounding meditation Dialogue about home practice Practice: sitting meditation about the program Satisfaction questionnaire How to facilitate our own practice Closing ritual | |

## S.7 Emotion regulation coding

**Figure S2.** Decision tree used as a reference for coding emotion regulation strategies.

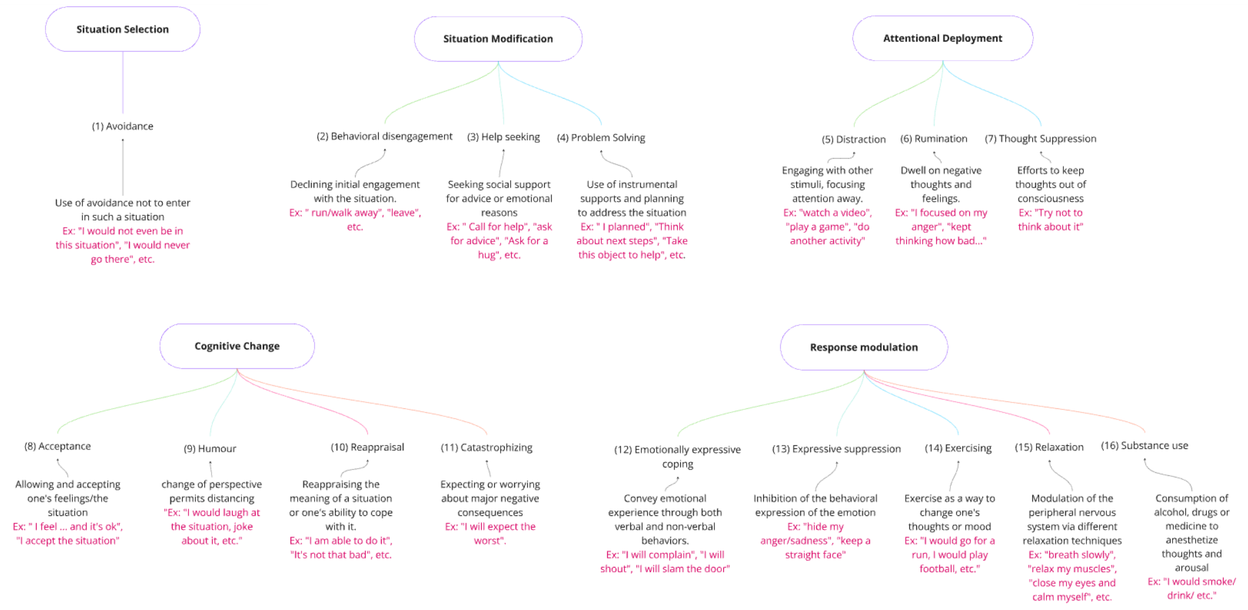


# References


Allen, V. C., et T. D. Windsor. 2019. « Age Differences in the Use of Emotion Regulation Strategies Derived from the Process Model of Emotion Regulation: A Systematic Review ». *Aging & Mental Health* 23 (1): 1- 14. https://doi.org/10.1080/13607863.2017.1396575.

Carthy, Tal, Netta Horesh, Alan Apter, Michael D. Edge, et James J. Gross. 2010. « Emotional Reactivity and Cognitive Regulation in Anxious Children ». *Behaviour Research and Therapy* 48 (5): 384- 93. https://doi.org/10.1016/j.brat.2009.12.013.

Fitzmaurice, G.M., N.M. Laird, et J.H. Ware. 2004. « Applied longitudinal analysis ». *John Wiley & Sons, Hoboken*.

Goubet, K. Elise, et Evangelia G. Chrysikou. 2019. « Emotion regulation flexibility: Gender differences in context sensitivity and repertoire ». *Frontiers in Psychology* 10. https://doi.org/10.3389/fpsyg.2019.00935.

Gross, James J. 1998. « The Emerging Field of Emotion Regulation: An Integrative Review ». *Review of General Psychology* 2 (3): 271- 99. https://doi.org/10.1037/1089-2680.2.3.271.

Gross, James J. 2014. « Emotion regulation: Conceptual and empirical foundations ». In *Handbook of emotion regulation, 2nd ed*, 3- 20. New York, NY, US: The Guilford Press.

Koole, Sander L. 2010. « The psychology of emotion regulation: An integrative review ». In *Cognition and emotion: Reviews of current research and theories*, 128- 67. New York, NY, US: Psychology Press. https://doi.org/10.4324/9780203853054.

McHugh, Mary L. 2012. « Interrater Reliability: The Kappa Statistic ». *Biochemia Medica* 22 (3): 276- 82.

Naragon-Gainey, Kristin, Tierney P. McMahon, et Thomas P. Chacko. 2017. « The Structure of Common Emotion Regulation Strategies: A Meta-Analytic Examination ». *Psychological Bulletin* 143 (4): 384- 427. https://doi.org/10.1037/bul0000093.
